# Fritz Schaudinn

**Published:** 1908-07

**Authors:** 


### FRITZ SCHAUDINN.

An interesting review of the work of Schaudinn appeared in the June issue of the Johns Hopkins Hospital Bulletin. Schultz gives a short biography of Schaudinn, the discoverer of the organism now generally conceded to be the cause of syphilis, and as his work in zoology and protozoology has been of such fundamental importance we will reprint a few of the facts concerning his work as presented by Schultz.

Fritz Richard Schaudinn was born on the nineteenth of September, 1871, at Roesiningken, a village of East Prussia. He obtained his degree of Doctor of Philosophy on the third of March, 1894. He died on the 22nd of June, 1906. Conserning his youth little need be said. Into his short life of less than thirty-five years, into a working period of twelve years, there was crowded a wealth of work, which, judged by the standard of quality alone, must stamp the author an indefatigable worker. Judged by the higher standard of quality, his work makes of him a genius, one of those truly wonderful master-minds that appear only once in a long period of time. When he died, scientific medical research lost its most brilliant exponent, a man destined by training and ability to carry us far along certain lines of work. Although much progress will undoubtedly be made along the paths marked out by him, the fullest possible measure of that progress will not be realized until there shall appear another with the trained technique, the keen observation, and the intellect of Fritz Schaudinn.

His observation, in 1894, that an adult protozoan with the organelles characteristic of one class may produce young with organelles supposed to be specific of an entirely different class, may be considered the beginning of Schaudinn's long series of additions to our knowledge of the protozoan life cycle.

He did extensive work on the malarial parasite, ankylostomiasis and amebic dysentery. Much confusion existed as to the part played by the ameba coli and it remained for him to describe the difference in at least two species of amebae and differentiate that associated with dysentery from the species found in the healthy intestine. What would have been the ultimate results of Schaudinn's labors, had he lived to complete them, cannot be imagined until they shall be taken up by another equally gifter.

